# Case of Diabetes Mellitus, in an Advanced Stage, Treated

**Published:** 1861-02

**Authors:** J. W. McKinney

**Affiliations:** Camargo, Ill.


### CASE OF DIABETES MELLITUS

### IN AN ADVANCED STAGE, TREATED.

BY J. W. McKINNEY, M. D., of Camargo, Ill.

While residing in Centralia, Ill., I was called, on the morning of the 23rd February, 1858, to see a step-son of Mr. S., a lad of about sixteen years old, who, it was stated, had been in bad health during the past eight months. On visiting this patient, I found him greatly emaciated, so much so that he could not sit upon a chair but a few minutes without an attack of syncope. A shrunken and contracted appearance marked his features. His pulse was frequent and feeble; tongue of a red glazed appearance, with a clammy gum on the lips and teeth, and an offensive sweetish odor of the breath. Hearing quite obtuse, which was first observed about three months previous to the above date, since which time it has gradually increased, requiring now to be spoken to in a very loud tone to understand what is said to him. Constant thirst, with an insatiable appetite for food; has been in the habit, daily, of drinking from one to two gallons of water. In the amount of food he has been somewhat restricted for the last two months, yet by no means sufficiently so as to quality. On inquiry, I learned he usually passed from one to three gallons of urine every twenty-four hours, which, for a week previous to my visit, had been attended with more or less pain along the course of the urethra, each time he had occasion to evacuate the urinary bladder.

The urine was of a pale greenish-yellow color, and of a faintly-sweet odor. On putting two fluid ounces of this urine into a loosely covered vessel, and applying a gentle heat, sufficient to evaporate the watery portion, I obtained of solid ingredients in a granulo-crystaline form *five drachms and four-*

*teen grains* (apothecary's weight) as a residue. The odor given off while this evaporating process was going on, was entirely destitute of the peculiar smell of urine, but, in stead, gave off the strong odor of boiling molasses. The taste was slightly alkaline, with a strong saccharine preponderance.

Having heretofore regarded this disease in its advanced stage as incurable, I could but look upon this case as a hopeless one. Accordingly, I gave the parents of the boy to understand what I considered to be the most probable result in the case—that we could have but little hope of his recovery; but notwithstanding this, I would put him on a course of diet and medicine that I wished strictly to be carried out, assuring them at the same time that all hope for his recovery depended upon the faithfulness with which they would adhere to the treatment. After being assured that all our directions would be strictly persevered in, I directed him to be put on an almost exclusive *animal diet*, composed of boiled beef or mutton, with bread made of wheat flour from which all the starch was to be carefully washed, and then "made light" before baking. Of this, he was permitted to take for breakfast and dinner, one quarter pound of the beef or mutton and four ounces of the bread at each meals. For supper, two poached eggs, with a little butter to make palatable, and the same amount of bread as at each of the other meal. In addition to this, he was ordered to take one half pint equal parts fresh milk and lime-water at each meal; besides this, his drink not to exceed one pint of water per day.

Ordered him to take of the Syr. of Iod. Ferri 1½ drachm, with 25 gtt. Laudanum, at 12 A. M. and 6 in the evening.

Feb. 14th—Patient had rested tolerably well during the afternoon and night; had urinated five times during night, with a slight diminution in quantity of urine voided; complains considerably of pain in the urethra when urinating. Ordered Bi-Carb. Soda 10 grs. with 20 grs. Calomel to be given at once, and if the bowels are not operated in six hours, to give a dose Oleum Ricini. As soon as bowels were opened, to give Carb.

Ferri 8 grs., Quinine 3 grs., with 20 gtt. Laudanum every six hours.

Feb. 25th—Calomel had operated freely without taking oil. Stomach very much nauseated before calomel operated; very thirsty during night, with slight increase in the amount of urine passed. Tongue moist, but coated with a whitish fur; clammy accumulations on the lips and teeth; pulse feeble and frequent.

Ordered a continuation of the Carb. Ferri and Quinine, as on the previous day, alternated with the Syr. Iod. Ferri 1½ dr. every three hours. The diet to be strictly adhered to as directed at my first visit, with no more than a pint of water in addition to the milk and lime-water as drink in twenty-four hours.

Feb. 27th—Patient much the same as at last visit, with less thirst. Continue treatment.

March 1st—Patient seems to be improving. Ordered a discontinuation of the Syr. Iod. Ferri, and about 4 grs. Carb. Ammon. to be added to Quinine and Iron given every 6 hours. Laudanum to be omitted in all the portions, except in the evening.

March 4th—A decided improvement is apparent in every condition of the patient this morning. Continue treatment, omitting the dose at midnight. Suffice to say that this patient gradually improved under the above treatment, which was faithfully kept up till the 20th of May, at which time he was entirely rid of all diabetic symptoms, and seemed to be in the enjoyment of good health.

